# What Anæsthetic Shall We Use?

**Published:** 1878-03

**Authors:** Julian J. Chisholm

**Affiliations:** Professor of Eye and Ear Diseases, University of Maryland, and Surgeon in charge of the Baltimore Eye and Ear Institute.


### ARTICLE II.

### *What Anæsthetic Shall We Use?*

BY JULIAN J. CHISHOLM, M. D.

Professor of Eye and Ear Diseases, University of Maryland, and Surgeon in charge of the Baltimore Eye and Ear Institute.

Surgeons in all parts of the world have been much exercised of late over this most important subject, and a question of greater interest to suffering humanity it would be difficult to frame. For the past thirty years anæsthetics have been in use, and millions who have been operated upon under their benign influences attest the inestimable value of this greatest of modern discoveries. Ether, although the first introduced, had made but little headway, when Simpson, in 1847, gave chloroform to the world. It met with immediate acceptance from professional men; and within a very few months there was scarcely a surgeon of note in either hemisphere who had not used it, and extolled it in the strongest expressions of his mother tongue.

From its universal adoption the inhalation of chloroform became the precursor of every serious operation. Those who enjoyed with it quiet sleep, and who without it would have experienced intense suffering, were soon numbered by hundreds of thousands. Ever recurring wars, with thousands of wounded, gave surgeons an opportunity of using chloroform on a very large scale, which only added to the well-deserved reputation which it seemed to have enjoyed from its very introduction.

There was but one single alloy in this general jubilee. Now and then some patient would die when chloroform had been inhaled. In former times such fatal accidents on the operating table were common enough to every surgeon of large experience; but now, since the introduction of chloroform, there was an uncomfortable suspicion that in some way the inhalation was to be blamed for these fatalities; for under its life-saving influences nobody ought to die. With this impression once excited, each accident as reported and copied from journal to journal and from newspaper to newspaper frightened the public, and slowly undermined that confidence which surgeons had previously had in this anæsthetic. A strong desire was expressed to discover some kindred agent which would establish anæsthesia without danger.

For many years the ether anæsthetic had been abandoned, and was nearly forgotten, chloroform having superseded it and driven it out of use. It was now remembered that so far very few deaths had been attributed to ether, and hence this older anæsthetic began with some to take the place that chloroform had so completely occupied. At this present time ether is nearly as much used as chloroform, its votaries being irregularly distributed. With many, chloroform holds its original undisputed sway; while with others sulphuric ether is extensively used. Now that ether is being more extensively employed, deaths under its administration are being more frequently reported, and hence the question which is forcing itself on the profession for solution: viz., which of these two anæsthetics is the safer, and by what safeguards can their administration be surrounded?

The many substances for anæsthetic purposes which have been brought to the notice of surgeons in the last few years have had but a very ephemeral existence; and with the exception of nitrous oxide gas, which dentists find so useful for their momentary operations in teeth extracting, all have passed from general use. Chloroform and ether alone remain as giants combatting for superiority.

That death occasionally occurs during the administration of both ether and chloroform, there can be no question. That deaths have occurred from the inhalation of either of these potent agents, even when the purest drug had been obtained and the inhalation most carefully administered, must be equally admitted. That deaths are numerous when either of these fluids are carefully administered no one well informed believes. That deaths are often wrongfully attributed to both of these anæsthetics, every one must acknowledge; for it is found very convenient to put the shortcomings of surgeons upon these agents, so that in common with the mal-administration of anæsthetics—extreme protraction of operations, so exhausting to patients—clumsiness in the surgical manipulation, with consequent loss of blood—temerity in undertaking operations, in which, from their magnitude, no reasonable success should have been expected, are disowned in the face of the inhalation, to which death is often conveniently attributed, even when the patient dies, many hours after the operation, from causes which would have been properly named prior to the discovery of anæsthetics.

I have seen anæsthetics administered carefully and carelessly, boldly, cautiously and timidly, by those well instructed in their use, and by others totally ignorant of their action. I have seen instances in which the most serious accidents might have happened, during the inhalation, had not steps been taken to prevent them. I propose in brief to analyze those cases, amounting to many thousands which have come under my own observation, and from their careful consideration deduce rules for the guidance of the surgeon in the administration of anæsthetics, by following which safety and success in the use of these most valuable agents can be secured.

For successful inhalation, much will depend upon the mode of offering the anæsthetic to the patient. The method adopted for administering anæsthetics is simple, and is the one in general use. Should I have control of the patient for twenty-four hours before I operate, I always insist upon a fast for at least six hours prior to the inhalation. This rule, however, I am compelled to break daily. I use anæsthetics extensively in my office practice to aid in diagnosis in the ophthalmic diseases of irritable and fretful children, as well as in operations at the Eye Clinic, which, being held at one o'clock in the day, immediately after the midday meal of the working classes, often compels me to administer the anæsthetic a very short time after a full meal has been taken. Beyond the vomiting, which is very annoying to the operator, and delays the speedy completion of the surgical procedure, I have seen no bad result from it. The precaution of loosening the clothing, especially that encircling the throat, chest and abdomen, so as to facilitate respiration, I never omit.

*With adults a drink of whiskey precedes the inhalation always.* Children bear anæsthetics so uniformly well that in my experience no such precaution is needed with them. A towel many times folded and formed into a hollow cone *with open top* if chloroform is used, and *closed top* if ether is to be administered, makes the very best inhaler that I am acqainted with, and should be the one in universal use. A thick towel can always be obtained, can usually be had clean, and will permit the more or less ready passage of air (as folded for the special anæsthetic to be used,) without which neither chloroform nor ether can be administered with safety. In my operating case I carry several long, stout pins, known as shawl pins, for the purpose of pinning together the ends of the folded towel in cone form. These simple instruments I find equally valuable with any in the operating case.

In using Ether, the cone with closed apex, having its cavity well soaked with the fluid, is placed directly over the nose and mouth of the patient and held firmly upon the face, notwithstanding the struggles and cries for breath, until the patient becomes prostrate and insensible. While the patient is struggling to escape from what he conceives to be an attempt to strangle him, be careful to keep only the free base of the folded towel pressed upon the face, leaving the mouth and nose free in the ample cavity of the cone, which pressure has not flattened or in any way interfered with. Should the cone be flattened against the patient's nose, very ugly symptoms of suffocation may be brought about, not by the ether, but by the towel. With chloroform such a procedure as the above described mode of administering ether would properly be called a very dangerous mal-administration, and deserving of severe censure.

Chloroform must be *slowly* inhaled without causing any suffocating feelings whatsoever. Into the cone, *with both base and apex open*, is poured a small quantity of chloroform. The towel is then held at some distance from the nose and slowly approached as the effects of the drug are experienced, until the base of the cone reaches the face about the time when the patient is half anæsthetized. The upper end of the cone is always carefully kept open for the free admission of air. By commencing the inhalation with a very dilute chloroform vapor no discomfort or suffocation is experienced by the patient, and sleep soon begins to mark its approach.

Another instrument, which should belong to the operating case of every surgeon, is a broad fenestrated or ring forceps, by which the tongue can be seized without injury and forcibly drawn out without fear of slipping away or tearing into its substance. I consider this instrument an essential to the surgeon who uses anæsthetics. There is a condition often met with during the early stages of inhalation in which the irritated pharyngeal muscles draw the tongue backwards, so as to seriously impede respiration. The free use of the forceps for drawing out the tongue and reopening the air passage is all-important at this period, as it removes one of the sources of danger.

During the inhalation I watch the face more than I do the pulse, although the face, the chest movements and the pulse are all kept under constant observation. The color and expression of the face will always be a sure index of the working of the heart and lungs. As soon as sleep begins to show itself, I take away the pillow from the head of the patient, so as to insure a horizontal decubitus, which would often be seriously objected to by the patient during the sensible condition. Should vomiting show itself, the patient is immediately turned over on the side until emesis is accomplished. The anæsthetic is then resumed, although retching may still continue. I find the continued inhalation the best method for checking the involuntary contraction of the abdominal muscles.

I continue to administer the anæsthetic until I find the reflex action suspended, as expressed by absence of contraction in the lids when the eyeball is touched. Then I consider the patient ready for operation, and in a safe condition to have it successfully performed. The patient lies in deep sleep, undisturbed by any peripheral irritation. The heart and lungs, through their own nerve centres, continue to work harmoniously, uninterfered with by reflex agencies, transmitted as they would otherwise be from the peripheral nerves through the cerebro-spinal axis to these special nerve centres. While the patient escapes all pain, and the surgeon avoids all the inconvenience of movements, the patient lies in motionless sleep, undisturbed by the surgeon's knife, all the faculties essential to life being in full operation. *This is the condition which I recognize as full and safe anæsthesia.* Less than this would permit dangerous and even fatal reflex complications; more than this would be extreme narcotism, which, by suspending the influence of the nerve centres, would stop the working of the organs—the heart and lungs—which these centres, when in good order, excite. Fortunately it would require much more of the anæsthetic to bring about this suspension of the vital function—a degree of narcotism which should justly be called an overdose. Between *this excessive narcotism and the safe and thorough anæsthesia there is a broad gulf, which only the very rash and ignorant are likely to bridge.* When the patient is fully anæsthetized, the inhalation is stopped, to be renewed from time to time if the operation be tedious and signs of returning consciousness make themselves apparent.

[TO BE CONTINUED.]

